# Perception of psychiatry among non-psychiatric physicians: a tunisian study

**DOI:** 10.1192/j.eurpsy.2023.1165

**Published:** 2023-07-19

**Authors:** M. Turki, F. Sahnoun, A. Samet, A. Guermazi, M. Ksibi, S. Ellouze, N. Halouani, J. Aloulou

**Affiliations:** Psychiatry B Department, Hedi Chaker University hospital, Sfax, Tunisia

## Abstract

**Introduction:**

Psychiatry is often perceived “different” by other medical professionals as well as by the general population. This perception of “difference” may give rise to stigma toward both patients with psychiatric disorders and mental health professionals.

**Objectives:**

The aim of this study was to investigate the attitudes of non-psychiatric and their perception of psychiatry and mental disorders.

**Methods:**

It was a cross-sectional, descriptive and analytical study, conducted among Tunisian undergraduate and graduate non-psychiatric physicians. Data were collected during September and October 2022, through an anonymous online questionnaire, spread throughout social media (Facebook), using the Google Forms® platform. We used the “Attitude toward Psychiatry-30” (ATP-30).

**Results:**

A total of 168 participants completed the questionnaire. Among them, 81 (48,2%) were undergraduate and 87 (51,8%) were graduate doctors. Their mean age was 26,4± 4.4 years, with a sex-ratio (F/M) of 3.4.

Among doctors, 79,2% had overall favorable attitudes toward psychiatry but only 38,2% among the undergraduate considered psychiatry as a potential career choice.

Psychiatry was considered as an unscientific and imprecise specialty by 20,3%; while 35,7% considered it as the least exciting.

The total score ATP-30 increased significantly with age (p= 0.023). It was significantly higher in those with psychiatric history (p=0.01).

**Conclusions:**

Our study showed a dissonance between favorable perception of psychiatry and the choice of psychiatry as a potential career. Therefore, it is crucial to identify factors that potentially account for this dissonance and enhance enthusiasm among undergraduate doctors as the shortage of psychiatrists may influence mental healthcare.

**Disclosure of Interest:**

None Declared

